# Mechanical Stability of Nano‐Coatings on Clinically Applicable Electrodes, Generated by Electrophoretic Deposition

**DOI:** 10.1002/adhm.202102637

**Published:** 2022-10-13

**Authors:** Vaijayanthi Ramesh, Nadine Stratmann, Viktor Schaufler, Svilen D. Angelov, Ilona D. Nordhorn, Hans E. Heissler, Ricardo Martínez‐Hincapié, Viktor Čolić, Christoph Rehbock, Kerstin Schwabe, Uwe Karst, Joachim K. Krauss, Stephan Barcikowski

**Affiliations:** ^1^ Institute of Technical Chemistry I University of Duisburg‐Essen and Center for NanoIntegration Duisburg‐Essen (CENIDE) 45141 Essen Germany; ^2^ Department of Neurosurgery Hannover Medical School 30625 Hannover Germany; ^3^ Institute of Inorganic and Analytical Chemistry University of Münster 48149 Münster Germany; ^4^ Electrochemistry for Energy Conversion Max‐Planck‐Institute for Chemical Energy Conversion 45470 Mulheim an der Ruhr Germany

**Keywords:** biocompatibility, biomaterials, colloids, deep brain stimulation, impedance, laser ablation in liquids

## Abstract

The mechanical stability of implant coatings is crucial for medical approval and transfer to clinical applications. Here, electrophoretic deposition (EPD) is a versatile coating technique, previously shown to cause significant post‐surgery impedance reduction of brain stimulation platinum electrodes. However, the mechanical stability of the resulting coating has been rarely systematically investigated. In this work, pulsed‐DC EPD of laser‐generated platinum nanoparticles (PtNPs) on Pt‐based, 3D neural electrodes is performed and the in vitro mechanical stability is examined using agarose gel, adhesive tape, and ultrasonication‐based stress tests. EPD‐generated coatings are highly stable inside simulated brain environments represented by agarose gel tests as well as after in vivo stimulation experiments. Electrochemical stability of the NP‐modified surfaces is tested via cyclic voltammetry and that multiple scans may improve coating stability could be verified, indicated by higher signal stability following highly invasive adhesive tape stress tests. The brain sections post neural stimulation in rats are analyzed via laser ablation‐inductively coupled plasma‐mass spectrometry (LA‐ICP‐MS). Measurements reveal higher levels of Pt near the region stimulated with coated electrodes, in comparison to uncoated controls. Even though local concentrations in the vicinity of the implanted electrode are elevated, the total Pt mass found is below systemic toxicologically relevant concentrations.

## Graphical abstract

The mechanical stability of surface modified neural electrodes via electrophoretic deposition using laser‐generated platinum nanoparticles is evaluated. In vitro stability tests show the lowest impedance increase after agarose gel test. After in vivo deep brain stimulation (DBS) in rats, the amount of biodispersed platinum is higher in coated electrodes though being fourfold lower than toxicologically relevant systemic concentrations.

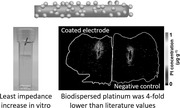

## 1 Introduction

Neural electrodes are employed for various treatments including deep brain stimulation (DBS) in Parkinson's disease, epilepsy, depression, deafness, spinal cord injuries, blindness, advanced tremors, etc.^[^ ^1^, ^2^ ^]^ Although these electrodes have long been used in clinics, a drawback lies in the increased electrode impedance (*Z*) due to gliosis, reducing the efficiency of stimulation/recording.^[^ ^3^ ^]^ To address this issue, researchers try to increase the electrode's electrochemical surface area (ECSA) utilizing various surface modification techniques.^[^ ^4^, ^5^, ^6^, ^7^, ^8^, ^9^ ^]^

Platinum (Pt) based electrodes are considered to be one of the best materials to act as a neural implant.^[^ ^10^ ^]^ Their surfaces have been widely modified using metals, metal oxides, conductive polymers and nanoparticles, for improving the electrochemical characteristics.^[^ ^11^, ^12^, ^13^, ^14^, ^15^, ^16^ ^]^ Established techniques used to modify platinum electrode surfaces include anisotropic etching,^[^ ^17^, ^18^ ^]^ two‐photon lithography,^[^ ^19^ ^]^ laser‐induced forward transfer (LIFT).^[^ ^20^ ^]^ as well as focused electron/ion beam induced deposition (FEBID/FIBID).^[^ ^21^, ^22^ ^]^ Particularly, studies, where the coating and the implant material are identical, are highly interesting, as they do not adversely affect the biocompatibility of the device and clinical approval is less complicated. However, if the coating delaminates from the implant surface and gets released into the organism, these advantages become invalid. Thereto, it is highly important to guarantee that the coatings are mechanically stable under in vivo conditions. Boehler et al. electrodeposited Pt on Pt—Ir electrodes and observed stable nanostructured coatings after 1 billion stimulation pulses that were biocompatible in vivo and efficient during stimulation.^[^ ^23^ ^]^ In another study, they deposited Pt nanograss on Pt microelectrodes through a chemical reduction method, which resulted in an increased ECSA and reduced *Z* of about two orders of magnitude.^[^ ^6^ ^]^ Pt—Ir has been electrodeposited on rectangular Pt cochlear electrodes^[^ ^24^ ^]^ and Pt—Ir microwire arrays^[^ ^25^ ^]^ resulting in a 91–93% reduction of polarization *Z* and low noise, high signal‐to‐noise ratio, and low *Z*, respectively. Zátonyi et al. deposited Pt black coatings on Pt ECoG microelectrode arrays (MEAs) and observed a reduction in thermal noise, enhanced signal‐to‐noise ratio, and increased ECSA.^[^ ^26^ ^]^ An alternate surface modification technique to nanostructure Pt‐based neural electrode surfaces is the electrophoretic deposition (EPD) of laser‐generated ligand‐free PtNPs.^[^ ^13^, ^15^, ^27^ ^]^ These purely electrostatically stabilized NPs are advantageous over their ligand‐coated counterparts, as they facilitate linear scaling of the deposition process concerning concentration and electric field strength, due to the absence of ligand‐induced electrostatic repulsion at the particle‐electrode interface.^[^ ^28^ ^]^ Using this method, we previously reported process parameter optimization for 2D target surfaces, and the in vivo functionality of 3D electrodes when coated with direct current (DC) EPD.^[^ ^15^ ^]^ In our recent work, we deposited PtNPs on 3D Pt—Ir surfaces and studied the electrode performances by applying DC and pulsed‐DC (PDC) electric fields,^[^ ^14^ ^]^ and further evaluated the impact of the solvent (ethanol–water mixtures) on coating quality.^[^ ^16^ ^]^ However, for a coating aimed at clinical application, its mechanical stability is one of the most critical factors to be studied. If the developed coatings are not stable enough, the detached particles may undergo biodispersion inside the body after implantation and can become toxic to the local tissue. Furthermore, coating delamination frequently goes along with impairment of electrode functionality. Boehler et al. chemically deposited nano‐Pt on Pt microelectrodes and tested the coating stability by dipping the modified electrodes in a 0.6% agar dummy. They found that the charge storage capacity (CSC) decreased by 4% and *Z* increased by 6%.^[^ ^7^ ^]^ Pt black coatings were deposited on various neural electrode surfaces, after which their durability was studied via ultrasonic agitation. Strong forces generated by ultrasonication deteriorated the electrochemical properties of Pt black modified surfaces: 77% decrease in ECSA,^[^ ^29^ ^]^ 21%,^[^ ^30^ ^]^ and 20%^[^ ^31^ ^]^ loss in CSC, and 63% increase in *Z*.^[^ ^8^ ^]^ Minev et al. produced stretchable composite coating (mixture of Pt powder and silicone) on MEAs and tested the mechanical integrity using a tensile stretcher and found that the coatings deformed with the applied strain but retained their electrical contact with the MEAs.^[^ ^32^ ^]^ Wang et al. performed ultrasonic agitation of their Pt‐black:PEDOT/PSS coated microelectrodes inside agarose gel to simulate brain tissue micromotions. They found a *Z*‐increase of 9.5% after 100 min of mechanical agitation and hence concluded that the coatings were durable for long‐term usage.^[^ ^33^ ^]^ In addition, material adhesion tests were also performed on modified neural electrode surfaces following the American Society for Testing and Materials (ASTM D3359),^[^ ^34^ ^]^ wherein a pattern (usually an X or a crosshatch) was scratched on the coatings, after which a pressure‐sensitive adhesive tape was pressed on the pattern and removed at an angle to observe coating delamination.^[^ ^35^, ^36^, ^37^, ^38^ ^]^ Another important assay concept for evaluation of electrode mechanical stability is a standardized bending fatigue test, for example, based on a sliding‐plate assay, where correlations between bending angle and resistivity are recorded.^[^ ^39^ ^]^ Here, Kim et al. could show that in hybrid structures where the Cu thin films were deposited on polyimine supports, bending strain is a direct function of film thickness.^[^ ^40^ ^]^ However, this method is primarily applicable to flexible electrode thin films on polymer support and difficult to apply to free metal wires with micrometer diameter in direct contact with tissue.

Nevertheless, to the best of our knowledge, a comprehensive and exclusive study on the mechanical stability of neural electrode surface coatings with several complementary testing methods, and detailed electrochemical analysis, including correlation with in vivo stability is rare. In this work, we present a systematic investigation of the stability of PtNP EPD on 3D Pt—Ir (90:10) neural electrode surfaces. Pt—Ir wires were coated with DC‐ and PDC‐EPD and their coating stabilities were tested via three approaches: dipping in 0.6 wt% agarose gel, adhesion test, and ultrasonic agitation. In addition, the electrochemical stability of the coatings was evaluated by cycling the samples multiple times in cyclic voltammetry (CV). Please note that optimization of coating parameters has been addressed in previous studies. Here, we aimed to evaluate the quality of an EPD‐coating synthesized under conditions optimized for optimum performance (low impedance) based on the following previous works^,[^ ^14^, ^15^, ^16^ ^]^

**Figure 1:**
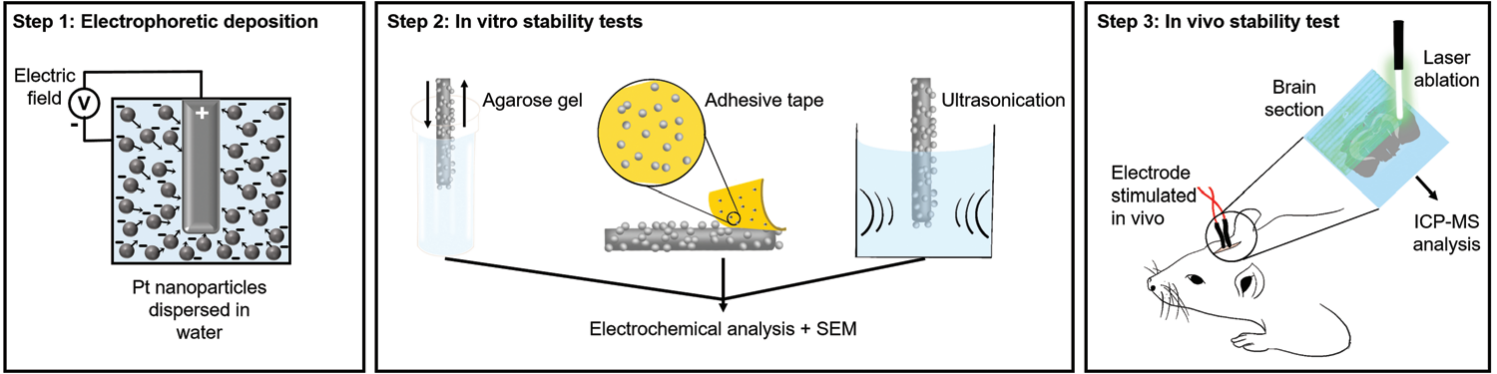
Schematic representation of the experimental workflow: Step 1 shows the EPD of negatively charged PtNPs on positively charged Pt neural electrode surfaces; Step 2 represents the three in vitro stability testing methods; and Step 3 shows the analysis of rat brain sections via LA‐ICP‐MS.

The surfaces were electrochemically characterized before and after performing stability tests via CV and electrochemical impedance spectroscopy (EIS). In addition, the rat brain slices post‐DBS were quantified for the presence of Pt using laser ablation‐inductively coupled plasma‐mass spectrometry^[^ ^41^ ^]^ (LA‐ICP‐MS) (**Figure** **1**). Furthermore, qualitative characterization of the coated surfaces was performed using scanning electron microscopy (SEM).

## 2 Results and Discussion

**Figure 2:**
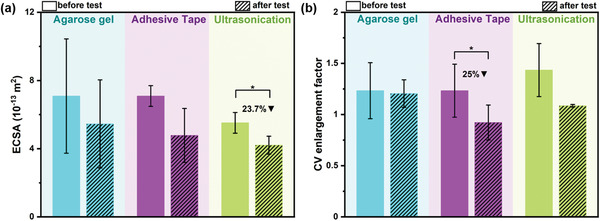
Electrode surface chemistry before and after mechanical stress testing. a) Average ECSA values of PDC‐coated neural electrodes, measured by applying 20 CV cycles (*N* = 4, *α* = 0.05). b) Average CV enlargement factor (CSC) values of PDC‐coated neural electrodes (*N* = 3, *α* = 0.05). Bar plots represent mean values and error standard deviations.

**Figure 3:**
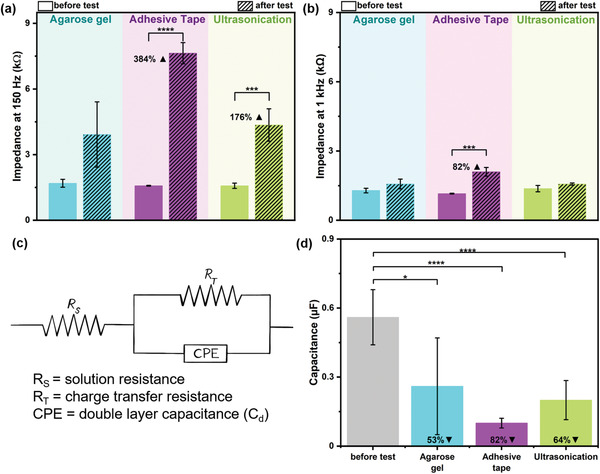
Average impedance values of PDC‐coated neural electrodes, before and after stability tests at a) 150 Hz and b) 1 kHz (*N* = 4, *α* = 0.05). c) Equivalent circuit model comprising an R(RC) circuit is used for fitting the EIS data. d) Average fitted capacitance values of the samples, before and after stability tests (*N* = 4, *α* = 0.05). Bar plots represent mean values and error standard deviations.

**Figure 4:**
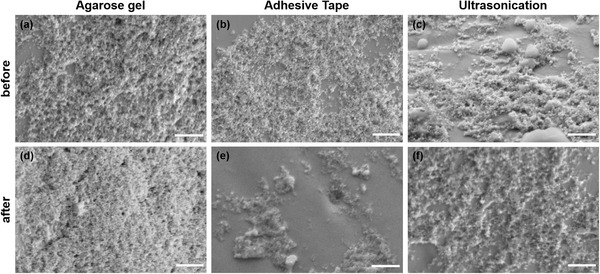
Exemplary SEM images from the sides of the PDC‐coated neural electrodes a–c) before and d–f) after the mechanical stability tests. Scale bars are 500 nm.

To evaluate the mechanical stability of EPD coatings, micro‐scale (76 µm diameter) Pt—Ir (Pt_90_Ir_10_ molar ratio) wire surfaces with dimensions and composition comparable to commercially available brain electrodes were modified using laser‐generated, ligand‐free colloidal Pt NPs. Particles were synthesized via a two‐step laser‐based approach starting with pulsed laser ablation of a Pt target in Milli‐Q water followed by a consecutive laser fragmentation in liquid (LFL) step for size control yielding a final mean particle diameter of 14 nm. Consecutively, the Pt NPs (100 µg mL*^−^* ^1^, pH11) were deposited on the wires by EPD utilizing DC (5 V cm*^−^* ^1^, 5 min) and PDC (5 V cm*^−^* ^1^, 1 µs period, 50% duty cycle, 10 min) electric fields as previously reported.^[^ ^14^ ^]^ The coating stability was assessed via three methods: dipping the coated samples in agarose gel (0.6 wt%), adhesion test using 3M Scotch tape, and ultrasonication in Milli‐Q water for 5 min. Before and after the mechanical tests, the samples were characterized using CV, EIS, and SEM. Since in our previous work, PDC‐EPD was reported to significantly reduce the neural electrode's *Z* compared to the DC‐EPD,^[^ ^14^ ^]^ we focused our study on the PDC‐EPD coatings while DC‐EPD results are presented in Section S4, Supporting Information. **Figure** **2** demonstrates the decrease in ECSA, **Figure** **3** shows the increase in *Z* and **Figure** **4** shows the neural electrode surfaces, after the stability tests performed on PDC‐coated samples. Their corresponding cyclic voltammograms and EIS spectra are shown in Figure S6, Supporting Information. To achieve sufficient signal stability all samples were cycled 20 times in the electrochemical setup before measurements.

From Figure 2a, it is observed that the ECSA of all the samples that underwent stability tests, decreased. Among them, the decrease in ECSA was the least in samples tested using an agarose gel. Similarly, the CSC also showed the least decrease in agarose gel compared to the other two tests (Figure 2b). Figure 3a,b shows the increase in *Z* values at both of the medically relevant frequencies after the stability tests, revealing an insignificant increase in *Z* after the agarose gel test at 150 Hz. It is well known from the literature that the ECSA and *Z* are inversely proportional to each other.^[^ ^8^, ^42^ ^]^ Therefore, the obtained results are in good agreement with each other. The significant levels of delamination, represented by *Z* increase, after adhesion test or ultrasonication are due to the high mechanical stress induced on the particle coatings, which causes them to peel off. During the agarose gel test, however, much lower friction forces of ≈5 µn were applied. Additionally, from the SEM images, it is clear that the adhesive tape removes more particles from the surface than ultrasonication (Figure 4e,f). This could be due to the distributed coatings produced by PDC‐EPD,^[^ ^14^ ^]^ which can be observed in Figure 4a–c. Here, since the NP coverage is less clustered in comparison to the DC‐EPD (Figure S5a–c, Supporting Information), the force generated by the adhesive tape was strong enough to remove a large number of monolayer particles from the surface than the force induced during ultrasonication, which probably has only removed the agglomerated particles that are loosely bound. SEM images from Figure 4 support this explanation, where after the tests the particles are not entirely removed from the electrode surfaces, however, more particles seem to have been removed after the tape test (Figure 4e). Boehler et al. found a 4% decrease in their CSC,^[^ ^7^ ^]^ however, our samples show no statistically significant decrease in CSC after the agarose gel test. Therefore, the mechanical stability tests confirm that the EPD‐generated coatings are more stable in an agarose gel (simulated brain density and viscosity) environment, compared to the other two in vitro stability tests.

**Figure 5:**
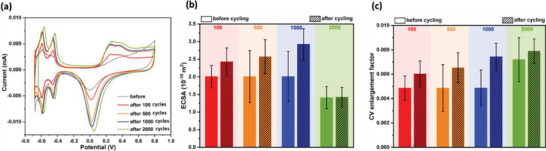
a) Exemplary cyclic voltammograms of PDC‐coated electrodes before and after electrochemical stability test, b) average ECSA values of the electrodes before and after electrochemical stability test (*N* = 3, *α* = 0.05), and c) average CSC values of the electrochemical stability tested electrodes (*N* = 3, *α* = 0.05). Bar plots represent mean values and error standard deviations.

**Figure 6:**
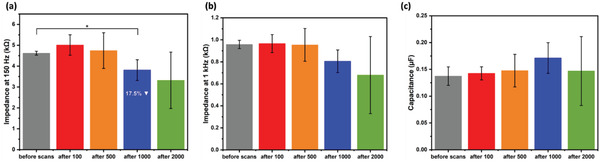
Average impedance values of electrochemical stability tested electrodes at a) 150 and b) 1 kHz. c) Average fitted capacitance values of the electrodes obtained after EIS equivalent circuit fitting. Bar plots represent mean values and errors standard deviations (*N* = 3, *α* = 0.05).

To investigate the electrochemical stability of the EPD‐generated coatings, Pt—Ir wires coated with Pt NPs using PDC‐EPD were scanned in CV for multiple cycles: 100, 500, 1000, and 2000. Before and after CV scanning, the *Z*‐values of the samples were measured. **Figure** **5** shows the CV analysis and **Figure** **6** shows the EIS analysis of the samples. The corresponding EIS spectra are shown in Figure S7a, Supporting Information.

Figure 5 shows that the ECSA and CSC of the samples gradually increase with an increasing number of CV cycles up to 1000 cycles. This finding may be attributed to the fact that repeated CV scanning of Pt surfaces in sulfuric acid helps to maximize the exposure of surface Pt sites for hydrogen adsorption,^[^ ^43^ ^]^ in turn activating the surface and increasing the ECSA and CSC^[^ ^43^, ^44^ ^]^ of the electrodes (Figure 5b,c). Interestingly, for longer cycling at 2000 cycles, the trend seems to decrease and no differences between the samples before and after cycling is available, which may point to more extensive particle delamination at a higher number of cycles, which seems to counter the observed activation effect at lower cycle numbers. EIS analysis on the samples before and after multiple CV cycles, reveals a significant decrease in *Z* at 150 Hz after 1000 cycles and no significant differences at longer cycling after 2000 cycles, which points to a saturation effect. (Figure 6a). At 1 kHz, the decreasing trend starts already after 500 cycles (Figure 6b), although not statistically significant, and here as well, saturation seems to occur after 2000 cycles. Upon fitting the EIS data with an equivalent circuit, the capacitance also shows an increasing though non‐significant trend after 1000 cycles and saturation at 2000 cycles. Wang et al. found that after 10 000 CV cycles the *Z* increased,^[^ ^33^ ^]^ representing coating instability. However, it should be noted that Wang et al. evaluated the performances of electrodes with a completely different design. In their case layered electrodes consisting of a platinum black/conductive polymer sandwich structure were analyzed, while in our work coatings composed of Pt‐electrode Pt‐nanoparticle metal‐metal interfaces were evaluated.

Our samples showed a statistically significant 17.5% reduction in *Z* after 1000 cycles at 150 Hz, revealing an improvement in their stability at intermediate cycles and saturation in impedance at 2000 cycles. Based on these findings it may be concluded that cycling a coated electrode in an electrochemical setup for 500–1000 cycles can improve certain properties like higher ECSA or lower impedance, though this effect is negated when more cycles are used, probably due to more pronounced coating delamination with more cycles.

**Figure 7:**
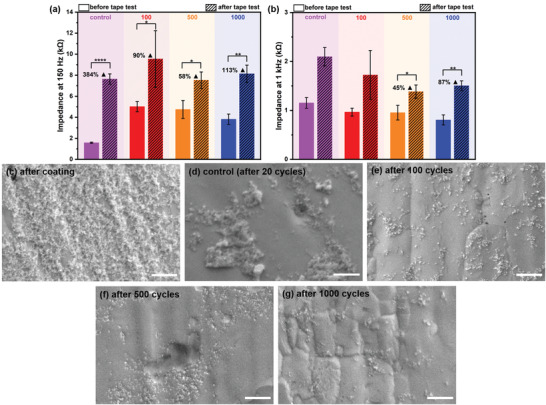
Average impedance values of the electrodes before and after adhesion test at a) 150 and b) 1 kHz, after electrochemical stability testing (*N* = 3, *α* = 0.05). The control samples are previously described in Figure 3, which were tested for adhesion with 20 previous CV scans. Bar plots represent mean values and error standard deviations. Exemplary SEM images of the PDC‐coated, adhesion tested samples: c) after coating; d) control (after 20 cycles); e) after 100 cycles; f) after 500 cycles; and g) after 1000 cycles. Scale bars are 500 nm.

To additionally test whether the extended CV cycling influences the mechanical stability of the deposited particles, adhesion tape tests were performed on the samples after CV cycling. **Figure** **7** shows the EIS analysis and SEM images, before and after the tape test, respectively. Please note that the controls used here are not derived from values without cycles as in Figure 6 (before scan) but had also undergone limited cycling (20 cycles) to allow comparability to previous stability assays. This fact accounts for inconsistencies in starting impedance at 150 Hz between Figures 6 and 7, though the massive differences occurring here cannot be explained based on the current experimental design.

Since the adhesion test induced the highest force on the particles and resulted in the highest levels of delamination previously, we were interested to investigate the strength of particle adherence after repeated CV cycles via the tape test. Figure 7a,b reveals a significant increase in *Z* after the test in all samples, at both frequencies with similar final impedance values independent of the previous cycling. However, the percentage increase in *Z* was lower in those samples that underwent multiple CV cycling. Also, the least increase in impedance can be observed after 500 CV cycles at both 150 Hz and 1 kHz. Figure 7c–g shows the corresponding coated surfaces after the tape test, indicating that the adhesive tape led to the expected pronounced delamination of the particles in the coatings, though particle detachment was not quantitative. Furthermore, multiple cycling before the tape test influenced the final morphology of the coating. Here more homogeneous coatings after multiple cycles were found pointing toward surface restructuring, while in the controls, coatings were primarily composed of multilayer assemblages though the images seem to indicate higher surface coverage. However, please note that in assemblage‐like multilayers, the contact between electrode and coating is less pronounced, which may explain why the final impedance in the multi‐cycled and control samples were similar even though surface coverage with particles in the controls was higher.

Therefore, for Pt‐based neural electrode fabrication, we demonstrate a surface coating technique using PDC‐EPD of laser‐generated Pt NPs, which decreases electrode *Z* significantly in comparison to their DC counterparts.^[^ ^14^ ^]^ Concerning the mechanical stability of these coatings, we note that additional multiple cycling of the electrode has a significant impact on coating stability with a minimum impedance change after 500 cycles. This went along with substantial differences in coating morphology, which retained assemblage‐based coatings in the controls, whereas more homogeneous coatings were found after continuous cycling. This could lead to an improved particle‐electrode electrical contact, lowering the overall impedance increase.

Another relevant kind of mechanical stress on a nanoparticle‐coated micrometer‐sized neural electrode would be bending. To examine this, we bent a 76 µm diameter Pt—Ir wire coated via PDC‐EPD with a tweezer assuming a final angle of ≈90°. The wires before and after the bending test were examined using SEM (Figure S9, Supporting Information). We could observe no further delamination or significant changes in nanoscale coating geometry due to macroscopic bending, which seems to indicate coating stability upon macroscopic electrode deformation.

**Figure 8:**
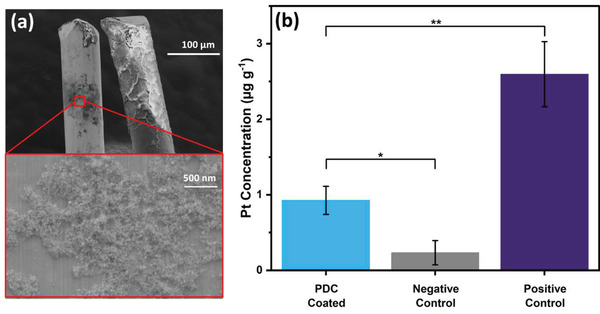
a) In vivo coating stability: SEM image of electrode coatings after 4 weeks in vivo stimulation in rat's brain. b) In vivo functionality of biodispersed Pt mass: average Pt concentration found in the brain slices stimulated with PDC‐coated electrodes (*N* = 3, *α* = 0.05), uncoated electrodes (*N* = 3, *α* = 0.05), and in the ex vivo brain slices injected with Pt NP colloid solution (positive control) (*N* = 2, *α* = 0.05). Bar plots represent mean values and error standard deviations.

**Figure 9:**
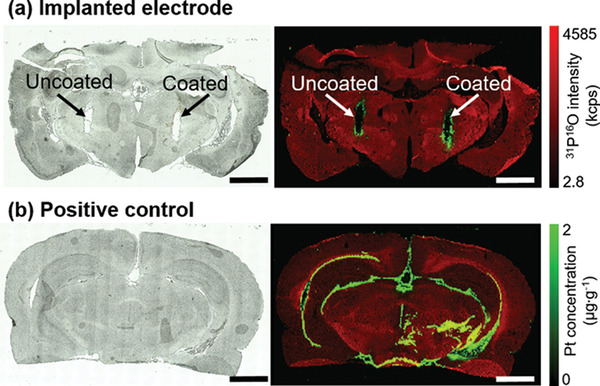
Pt biodistribution after 4‐week DBS in rat brain: a) optical microscopic and LA‐ICP‐MS overlay images of brain sections stimulated with uncoated and PDC‐coated electrodes; and b) Optical microscopic and LA‐ICP‐MS overlay images of brain sections injected with Pt NPs. Scale bars are 2 mm. In the overlay pictures, red signals represent the intensity of phosphorus, and green signals represent the Pt concentration.

To analyze the stability of coated NPs inside an in vivo environment, coating stability was evaluated by SEM after 4 weeks of in vivo DBS and the brain sections of rats that underwent 4‐week DBS as previously described in our work,^[^ ^15^ ^]^ which were mounted on glass slides, and Pt content in the tissue surrounding the implantation site was quantified using LA‐ICP‐MS. In this previous work, we already demonstrated a pronounced stabilization of impedance in vivo for 4 weeks, while the impedance in the uncoated controls increased in the corresponding period verifying the beneficial properties of the analyzed coatings. Hence, this work focuses on coating degradation and dissolution in vivo and does not aim to reproduce the functionality assays. Please note that the electrodes used for in vivo studies in this work were not pre‐treated by multiple cycling. **Figure** **8**a depicts SEM images of neural electrodes after removal from the rat's brain after a 4‐week stimulation period. While many of the electrodes were contaminated with organic residues (Figure 8a, right), a minor fraction of the examined electrodes remained uncontaminated (Figure 8a, left). Here the presence of a partially intact nanoparticle surface coating can be verified (Figure 8a) and qualitatively only minor differences between the coatings before stability tests (Figure 4, top row) and those after in vivo stimulation are observable. Based on this finding we conclude that neither the long‐term in vivo stimulation period nor the physical strain during electrode removal substantially degraded the electrodes’ coatings. Figure 8b shows the amount of Pt present in the stimulated brain region using PDC‐coated electrodes, uncoated negative control, and colloid injected positive control. Pt quantified in brain regions stimulated with coated electrodes was significantly higher than the negative control and significantly lower than the positive control. **Figure** **9**a represents the distribution of Pt inside the brain sections that were implanted with uncoated and PDC‐coated electrodes. The intensity of the green color (Pt) is higher in the coated side of the brain in comparison to the negative control (uncoated electrode). It should be noted that the insertion holes in Figure 9a are caused by the electrode housing. Due to the friction forces during implantation, Pt from the electrode tips could have diffused to the inner sides of the brain tissues revealing the presence of Pt. Furthermore, the green signals are the highest in the positive control (NP solution injected) brain slices (Figure 9b). Since the amount of NP injected in the positive control was higher (50 µL), it is likely that the colloidal NP solution migrated out of the tissue and entered the surrounding fluid, revealing the Pt signal in the arachnoidal space. Interestingly, Pt signals were distributed along the implantation tracks of the uncoated and PDC‐coated electrodes and not solely where the electrode tip was located. Robblee et al. have observed a similar behavior after stimulation of Pt electrodes and proposed diffusion processes and fluid exchanges as explanations.^[^ ^45^ ^]^ In the case of the PDC‐coated electrode, also a detachment of Pt NP from the surface during electrode insertion or removal could be an explanation, but this would not apply to the uncoated electrode. To further investigate these findings, two additional experiments were performed and the LA‐ICP‐MS results are presented in Figure S8, Supporting Information. By analyzing a brain sample with only steel casing implantation (without PtIr electrode) (Figure S8d, Supporting Information) a release of Pt from surgical equipment or an introduction of Pt from other sources than the implanted electrodes was ruled out. Furthermore, an unstimulated brain sample after short‐term implantation of a PDC‐coated electrode and an electrode immersed in Pt NP were analyzed (Figure S8b, Supporting Information). Here, Pt is detected for both electrodes revealing that Pt is also released from the electrodes without stimulation, while immersed and coated electrodes release about the same amount of Pt. This is surprising though probably attributed to an overlay of two effects: I) particle release from the immersed electrode is more likely as they are only loosely bound to the electrode; and II) particles from the PDC‐EPD‐coated electrodes are more tightly bound but on the other side higher total deposition yields are reached. Hence, the interplay between these effects will result in similar amounts of Pt release in both samples. Nonetheless, the release is significantly lower than in electrodes exposed to long‐term in vivo stimulation (Figure S8a, Supporting Information). Overall, the results suggest that the broader distribution of Pt after stimulation cannot be entirely attributed to the detachment of Pt NPs during electrode insertion and removal, but that stimulation leads to the dissolution of Pt from the electrodes, regardless of the coating. Please note that isolated Pt signals located millimeters away from the insertion site, for example, in Figure 9a, are either the electronic noise of the ICP‐MS system or artifacts from the laser ablation process of the tissue sample. In particular, the fact that these particles are isolated and no gradual Pt change is visible makes their transport by a biological process highly unlikely.

Pt NPs are widely used in various biomedical applications, such as for drug delivery, medical implants, cancer cell detection and many more, and their toxicological nature was investigated by some researchers.^[^ ^46^ ^]^ Adeyemi and peers administered Pt NPs (10, 50, and 100 mg kg*^−^* ^1^) orally in Wistar rats for 30 consecutive days, which resulted in organ weight alterations, inflammation‐induced lesions, and cellular degeneration.^[^ ^47^ ^]^ They performed another study, where the highest dosage was 50 mg kg*^−^* ^1^ body weight, which resulted in oxidative stress induction in rat plasma.^[^ ^47^ ^]^ In another study, Pt NPs (15 mg kg*^−^* ^1^) were introduced intravenously into BALB/c mice, resulting in acute hepatic injury along with increased levels of liver enzymes. However, NPs of 15 nm sizes showed no significant change in the enzyme levels.^[^ ^48^ ^]^ Yamagishi and peers also studied the effects of Pt NPs size via intraperitoneal injection and found that 8 nm particles did not cause nephrotoxic reactions.^[^ ^49^ ^]^ Various other studies confirm that the Pt NPs are non‐cytotoxic and can be used in anticancer therapies.^[^ ^50^, ^51^ ^]^ Pt NP solutions (1–20 µg ml*^−^* ^1^) were injected into chicken embryos and their results indicated no adverse effect on their growth and development.^[^ ^52^ ^]^ In a recent study, researchers found that the FDA‐approved Pt‐based drugs such as cisplatin turned into Pt NPs in vivo in human blood, and it was biocompatible and hindered the growth of chemotherapy‐resistant tumors.^[^ ^53^ ^]^ As our findings reveal the presence of 1 µg Pt per gram of brain, for a rat brain weighing an average of 2 g,^[^ ^54^ ^]^ ≈2 µg of Pt would have been biodispersed. In comparison to the total weight of a rat (≈335 g), ≈0.006 mg kg*^−^* ^1^ body weight of Pt would have been systematically released. As this is at least four orders of magnitude lower than any systemic Pt concentration used in the literature for biocompatibility assays, systemic adverse effects are unlikely, however high local concentrations in the brain are to be considered as well. Thereto, in our previous work, we studied the neuronal cell counts and glial scar formation around the implantation site after neural electrode removal and found no differences between coated and uncoated electrodes.^[^ ^15^ ^]^ However, in the course of potential clinical approval of the coated electrodes more sophisticated biocompatibility assays, for example, monitoring of inflammatory markers would be useful though these studies are beyond the scope of this work.

## 3 Conclusion

The mechanical stability of implant coatings greatly influences their applicability in vivo. Herein, we performed a surface modification technique, EPD of laser‐generated Pt NPs, on Pt‐based 3D neural electrode surfaces. Due to a lack of a comprehensive study investigating exclusively the mechanical stability of neural electrode nano‐coatings, we evaluated the same using agarose gel, adhesive tape and ultrasonication. EPD‐generated coatings were more stable in agarose gel (mimicking brain tissue) compared to the other two complementary methods and stability was retained during in vivo stimulation in rat brains. A subsequent electrochemical stability test through multiple CV cycling reveals a further, statistically significant, 17.5% reduction in *Z* after 1000 cycles probably due to surface restructuring and activation. However, more cycles negated these effects probably due to further delamination, pointing at an optimum intermediate cycle number for maximum performance of the electrodes. Further, quantification of Pt in brain sections after DBS via LA‐ICP‐MS reveals a significantly higher amount of Pt in the brain region stimulated with PDC‐coated electrodes, in comparison to that of the control region. However, the body mass‐specific Pt dose released was four times lower in magnitude than those reported in Pt‐nanotoxicity literature. Based on these results, we conclude that a good way to fabricate high‐performance Pt neural electrodes with the least coating delamination is to electrophoretically deposit laser‐generated PtNPs on their surfaces followed by cycling the substrates for 500–1000 times in CV, which additionally activates the surface Pt sites and in turn improves electrode performance. Therefore, the demonstrated coating fabrication and stability testing could be applied to all relevant 3D implant manufacturing, while particularly the “activation” of the nano‐coating by the cycling of the electrode in an electrochemical cell constitutes an underexplored approach to improve electrode performance and coating stability.

## 4 Experimental Section

### Nanoparticle Generation

Ligand‐free Pt NPs were synthesized in Milli‐Q water via laser processing in liquids as previously described elsewhere.^[^ ^14^ ^]^ In brief, a Pt bulk target (10 × 10 × 1 mm^3^) was ablated in Milli‐Q water using an Nd:YAG laser (Ekspla, Atlantic series, 10 ps, 1064 nm, 9.6 mJ, 100 kHz), and subsequently laser fragmentation in liquid environment (LFL) using a nanosecond laser (Innolas, Spitlight, 9 ns, 532 nm, 84 mJ, 100 Hz, 1.5 J cm*^−^* ^2^) in a passage reactor. This synthesis route yields particles with an average hydrodynamic diameter of 14 nm and a total mass concentration of ≈500 µg mL*^−^* ^1^. These concentrated colloids were diluted to a concentration of 100 µg mL*^−^* ^1^ using Milli‐Q water and their pH was adjusted to a value of 11 using NaOH solution (0.1 m), before EPD. The zeta potential value of the Pt NP colloids was determined to be −62 mV.

### Target Substrate Preparation

PTFE coated Pt—Ir (90:10) wires (Science Products GmbH, Germany) with a diameter of 76 µm were used as coating substrates for the stability tests. These wires had the same geometry and composition as the neural electrodes that were used for in vivo stimulations. The wires were cut into lengths of 20 mm and on both ends, the isolation was removed exposing 4 mm of Pt—Ir surface. One end of the wires was soldered to electrical plug pins and the other end was thoroughly rinsed with ethanol and introduced into Pt colloid for EPD. Bipolar electrodes for in vivo experiments were made of two parallel Pt—Ir (90:10%) wires insulated with Teflon (*d* = 0.0055″ with insulation and *d* = 0.003″ uninsulated; Science‐Products GmbH, Hofheim, Germany), placed in a 0.55 × 17 mm stainless steel tube cut from a 24G syringe needle. At the contact end, both wires were uninsulated leaving a 500 µm long bare surface with ≈250 µm intercontact distance. Contact pins were soldered to the other end. The electrode tip was cleaned and conditioned before coating by immersing it in 65% nitric acid for 15 min and then rinsing it thoroughly with distilled water. The first impedance measurement was done before coating, but after cleaning, to exclude changes induced by the cleaning procedure.

### Electrophoretic Deposition

A custom‐made EPD chamber for 3D wires was used for coating.^[^ ^15^ ^]^ For all the stability tests, the samples were coated using DC and PDC electric fields. The target substrates were connected to the positive pole of the electrical source and the surrounding metal counter electrode in the chamber was connected to the negative terminal. Pt colloid (600 µL) was filled into the chamber and the depositions were carried out. An electric field strength of 5 V cm*^−^* ^1^ was applied for both DC‐ and PDC‐EPD. In addition, for PDC‐EPD a period of 1 µs and pulse width of 500 ns was applied. The deposition time was set to 5 min for DC and 10 min for PDC‐EPD. The colloids were magnetically stirred during deposition to avoid sedimentation. Before and after coating, the colloids were characterized via UV–Vis extinction spectroscopy (Evolution 201, Thermo Scientific) in the wavelength range of 190–900 nm using a quartz cuvette with a path length of 10 mm to determine the deposited mass of NPs on each sample (Figure S2a, Supporting Information). The area under the curve (AUC) of the whole spectra was integrated and quantified against known Pt NP mass concentrations (Figure S2b, Supporting Information).

To characterize the coated surfaces, the electrode samples were mounted on aluminum holders and imaged via SEM (operating voltage: 5 kV, Apreo S LoVac, Thermo Fisher Scientific). It should be noted that the qualitative SEM analysis suffers from poor statistics and it is difficult to measure the same spot on the wires before and after stability testing. Therefore, after mounting the samples on aluminum holders, the images for “before stability testing” were performed. The mechanical stresses as described below were then applied to the samples, without removing them from the holders. Subsequently, the “after stability testing” images were taken. In this way, it could be made sure that the areas of the images are the regions where the stresses were applied.

### Mechanical Stability Tests

The mechanical stability of DC‐ and PDC‐coated wires were evaluated using three exemplary testing methods: dipping in agarose gel, ultrasonic agitation, and standard adhesion test (ASTM D3359–17). All the samples were characterized in CV and EIS, before and after stability testing. Additionally, SEM images were taken before and after the tests.

### Agarose Gel Test

DC‐ and PDC‐coated Pt—Ir wires were dipped into a simulated brain environment^[^ ^7^, ^55^ ^]^ (0.6 wt% agarose gel). For the gel preparation, agarose powder (0.6 g) was added to Milli‐Q water (100 mL) and stirred continuously at 95 °C until a transparent solution was obtained. The mixture was cooled down to 35 °C and transferred to plastic cuvettes and left to set overnight.^[^ ^56^ ^]^ The average dipping speed was calculated to be 2 ± 0.5 mm s*^−^* ^1^ and the friction force was calculated to be ≈5 µn using Stokes equation (compare Section S3, Supporting Information).

### Ultrasonic Agitation

EPD‐coated samples were ultrasonicated (PTIC‐30‐ES, ALLPAX GmbH & Co. KG, Germany) in Milli‐Q water at a frequency of 40 kHz for 5 min,^[^ ^8^, ^31^, ^32^ ^]^ after which they were characterized using CV, EIS and SEM. The energy applied to the samples was measured to be 864 kJ (refer to Section S3, Supporting Information).

### Adhesion Test

A slightly modified nano‐adhesion tape test was performed according to the American Society for Testing and Materials (ASTM D3359‐17).^[^ ^40^, ^41^, ^42^, ^43^ ^]^ 10 × 10 mm^2^ of 3M Scotch pressure‐sensitive tape was stuck on the coated wire surfaces and peeled off. Since the wires had a diameter of 0.076 mm and the coatings were presumed to be monolayer packing, an X was not scratched before applying the tape.

### Electrochemical Characterization

To evaluate the electrochemical properties of the depositions, the samples were characterized before and after stability tests using a three‐electrode setup potentiostat (VersaSTAT 3F, AMETEK Scientific Instruments, USA). A Pt wire was used as a counter electrode, Ag/AgCl or Hg/HgSO_4_ as reference electrodes and the coated or uncoated Pt—Ir wires as a working electrode. The electrochemical cell and all components in contact with the working electrode were soaked overnight in a mixture of ca. 3 g L^−1^ KMnO_4_ in 0.5 m H_2_SO_4_. After the permanganate solution is recovered, the pieces were rinsed with a diluted piranha solution and then boiled at least six times with ultrapure water. Here, a homemade RHE electrode, and Suprapur sulfuric acid were used.

### Cyclic Voltammetry

CV measurements were carried out in sulfuric acid (1 m). The potential window was set from −0.2 to 1.2 V versus RHE at a scan rate of 0.2 V s*^−^* ^1^. Before measurements, the electrolyte was purged with nitrogen for 45 min and thereafter for 5 min between consecutive measurements. Twenty cycles were performed for each sample and the ECSA was calculated from the voltammogram using the following equation:^[^ ^57^, ^58^ ^]^

(1) $$ECSA=Q_{H}/Q_{a}$$

where *Q*_H_ is the charge associated with hydrogen adsorption peak and *Q*_a_ = 210 µC cm*^−^* ^2^, the theoretical charge density of a polycrystalline Pt surface.^[^ ^43^, ^59^, ^60^, ^61^ ^]^

For testing the electrochemical stability of the coatings, the PDC‐coated samples were “cleaned” by sweeping for a varying number of CV cycles: 100, 500, 1000, and 2000. A three‐electrode setup potentiostat (VSP‐3e Potentiostat, BioLogic Sciences Instruments) was used. The potential window was set from −0.2 to 1.5 V versus RHE at a scan rate of 0.5 V s*^−^* ^1^. Sulfuric acid (0.5 m) was used as the electrolyte and purged with argon before the measurements. The CV enlargement factors were calculated to determine the CSC values, by integrating the area under the curve (AUC) of one full CV cycle.^[^ ^6^, ^62^ ^]^

### Electrochemical Impedance Spectroscopy

*Z* measurements were performed in potentiostatic EIS mode from 1 Hz to 100 kHz by applying an AC_rms_ value of 10 mV and using NaCl (0.9%) as the electrolyte. The measured values were plotted in Bode format (*Z*_mag_ versus frequency) and the *Z*_mag_ values at medically relevant frequencies (150 Hz and 1 kHz) were studied. In addition, the values of electrolyte resistance (*R*_S_), charge transfer resistance (*R*_T_), and double layer capacitance (constant phase element, CPE) were obtained by fitting the measured data to a one‐time constant equivalent circuit (OTC‐EC) model^[^ ^30^, ^63^ ^]^ (Figure 3c) using the software ZView (AMETEK Scientific Instruments, USA). The capacitance values were calculated from the obtained fitting results using the formula:^[^ ^64^, ^65^ ^]^

(2) $$\mathrm{Capacitance}=Rs^{(1-n)/n}{•Q}^{(1/n)}$$

where *Q* is the pseudo capacitance and *n* is the CPE order, both extracted from the CPE used in the EC.

### In Vivo Experiments

For the in vivo study electrodes were bilaterally implanted in the STN of 3 male Sprague‐Dawley rats as follows: Coated electrodes were always implanted in the left hemisphere; uncoated, in the right hemisphere. After two weeks of postoperative recovery, the animals received chronic electrical stimulation for 4 weeks (100 µA, 130 Hz symmetric, biphasic, and rectangular waves with a duration of 160 µs and polarization change after 80 µs were used. More details can be found in the authors’ previous work.^[^ ^15^ ^]^ After the stimulation period, the rats were deeply anesthetized with an overdose of chloral hydrate and transcardially perfused with paraformaldehyde (4%) solution. The brains were removed from the cranial cavity, placed in sucrose/phosphate‐buffered saline (30%) solution for at least 12 h, and cut on a freezing microtome (coronal plane) with a section thickness of 20 µm. For the positive control Pt NP microinjections, cadavers were fixed on the stereotaxic frame. After incision and defining the bregma, two burr holes were drilled bilaterally above the targets and 50 µL of Pt NP colloid (100 µg mL*^−^* ^1^) was injected into the subthalamic nucleus with a Hamilton syringe, using the following coordinates (in mm) relative to Bregma: anteroposterior: −3.8, mediolateral: ±2.5; dorsoventral: +8. The brain slices were mounted on glass slides and later analyzed via an LA‐ICP‐MS device.

### LA‐ICP‐MS Analysis

Experiments were carried out in accordance with the EU Directive 2010/63/EU for animal experiments, including approval by the Lower Saxony State Office for Consumer Protection and Food Safety (LAVES, AZ 18/2837). LA‐ICP‐MS measurements were carried out using an ImageBIO266 laser ablation system (Elemental Scientific Lasers, Bozeman, USA) coupled to an iCAP TQ ICP‐MS system (Thermo Fisher Scientific, Bremen, Germany) via a Dual Concentric Injector (DCI, Elemental Scientific Lasers). For the quantification of Pt in the brain tissue sections, an external calibration with matrix‐matched standards based on gelatin (10% w/w) was used.^[^ ^66^ ^]^ Ten calibration points in a concentration range from 0 to 80 µg g*^−^* ^1^ of Pt were prepared. The validation of Pt concentration in the gelatin standards was carried out by bulk analysis via ICP‐MS after digestion with nitric acid. Gelatin standards were cut into 20 µm thick sections with a cryomicrotome (Cryostar NX70, Thermo Fisher Scientific) and mounted on microscopic glass slides. Brain samples and gelatin standards were ablated in a line‐by‐line scan with a laser spot size of 60 µm, a scan speed of 180 µm s*^−^* ^1^, a space between the lines of 0 µm, and a laser repetition rate of 100 Hz. A helium gas flow of 1 l min*^−^* ^1^ was applied to transport the ablated material into the ICP‐MS. An additional argon gas flow of 1.1 l min*^−^* ^1^ was introduced after the ablation cell for plasma stabilization purposes. The ICP‐MS was equipped with a quartz injector tube with an inner diameter of 3.5 mm, a nickel sampler, and a skimmer cone. The ICP‐MS parameters were set to 1550 W RF power, 14 l min*^−^* ^1^ cool gas flow, and 0.8 l min*^−^* ^1^ auxiliary gas flow. The ICP‐MS was used in the triple quadruple mode with oxygen as a reaction gas. The isotopes ^31^P (detected as ^31^P^16^O), ^194^Pt, and ^195^Pt were monitored. ^195^Pt was used for data visualization, whereas ^194^Pt was used for validation purposes. It should be noted that the form of Pt (chemical species, NP, or ionic) could not be determined based on the results obtained. Bright‐field microscopic images were obtained before LA‐ICP‐MS analysis with a BZ‐9000 inverted fluorescence/bright field microscope (Keyence, Osaka, Japan). Data evaluation and visualization were performed using in‐house developed software (Robin Schmid, WWU Muenster, Muenster, Germany).

### Statistical Analysis

All data points are presented as mean values ± standard deviation the sample size was *n* = 3–4 and details for each experimental series are depicted in the figure captions. The statistical analysis was performed using OriginLab (v. 2020b) software. One‐way ANOVA evaluations were performed on the data with Tukey's test as a post‐hoc comparison. The *α* value was set to 0.05, and the levels of statistical significance are represented as *p ≤ 0.05, **p ≤ 0.01, ***p ≤ 0.001, and ****p ≤ 0.0001. All the presented data was tested for significance, but only those with a significant difference are explicitly depicted in the graphs.

## Conflict of Interest

The authors declare no conflict of interest.

## Supporting information

Supporting Information

## Data Availability

The data that support the findings of this study are available from the corresponding author upon reasonable request.
