# Supplementary material for: Mechanical Stability of Nano‐Coatings on Clinically Applicable Electrodes, Generated by Electrophoretic Deposition
Source: Adv Healthc Mater. 2022 Oct 13;11(23):2102637. doi: 10.1002/adhm.202102637 (PMC11468750; doi:10.1002/adhm.202102637)
Supplement: Supplementary file 1 — Supporting Information [file ADHM-11-2102637-s001.pdf]

ADVANCED HEALTHCARE MATERIALS

Supporting Information

for *Adv. Healthcare Mater.*, DOI 10.1002/adhm.202102637

Mechanical Stability of Nano-Coatings on Clinically Applicable Electrodes, Generated by Electrophoretic Deposition

Vaijayanthi Ramesh, Nadine Stratmann, Viktor Schaufler, Svilen D. Angelov, Ilona D. Nordhorn, Hans E. Heissler, Ricardo Martínez-Hincapié, Viktor Čolić, Christoph Rehbock, Kerstin Schwabe, Uwe Karst, Joachim K. Krauss and Stephan Barcikowski**

Supporting Information

Mechanical Stability of Nano-Coatings on Clinically Applicable Electrodes, Generated by Electrophoretic Deposition

Vaijayanthi Ramesh[1], Nadine Stratmann[1], Viktor Schaufler[1], Svilen D. Angelov[2], Ilona D. Nordhorn[3], Hans E. Heissler[2], Ricardo Martínez-Hincapié[4], Victor Čolić[4], Christoph Rehbock[1], Kerstin Schwabe[2], Uwe Karst[3], Joachim K. Krauss[2] and Stephan Barcikowski[1,*]

[1] Institute of Technical Chemistry I, University of Duisburg-Essen and Center for NanoIntegration Duisburg-Essen (CENIDE), Essen, Germany.

[2] Department of Neurosurgery, Hannover Medical School, Hannover, Germany.

[3] Institute of Inorganic and Analytical Chemistry, University of Muenster, Muenster, Germany

[4] Electrochemistry for Energy Conversion, Max-Planck-Institute for Chemical Energy Conversion, Muelheim an der Ruhr, Germany

S1. Particle size distribution

Figure S1 shows the average hydrodynamic number and weight distribution of laser fragmented platinum nanoparticles (Pt NPs) used for electrophoretic deposition (EPD).

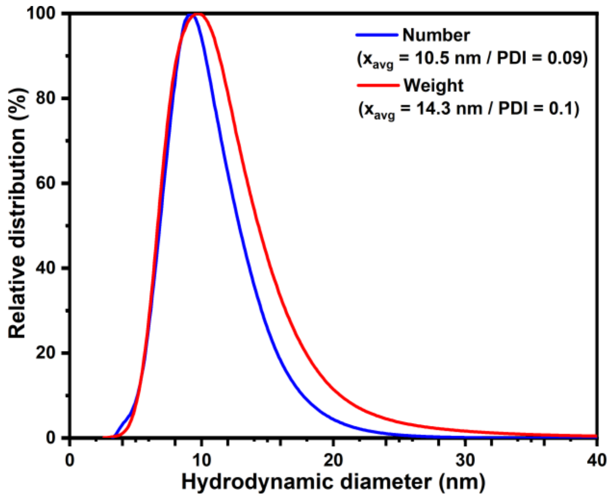

Figure S1: Average hydrodynamic size distributions of laser fragmented PtNPs, showing average diameters at 14 nm (weight %) and 10 nm (number %).

S2. Mass of Pt NP deposited on the samples

Mass deposited on the samples after direct current (DC) and pulsed DC (PDC) EPD was determined using UV-Vis extinction spectroscopy (Figure S2(a)). Area under curve (AUC) values of the spectra before and after EPD, were correlated with the known mass concentration gradients of Pt NPs (Figure S2(b)). The mass remaining in supernatants after EPD was subtracted from the mass of the colloid before EPD, to obtain the deposited mass on each

sample[1] (Figure S2(c)).

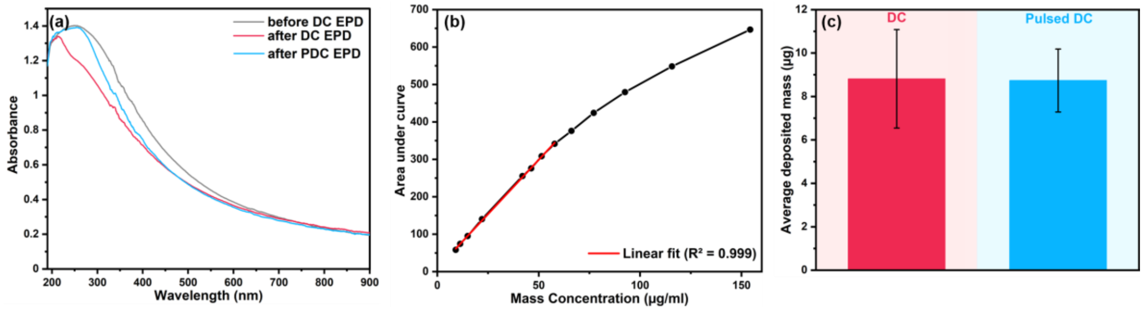

Figure S2: (a) Exemplary UV-Vis spectra of Pt NPs before EPD and after DC- and PDC- EPD, (b) mass concentration gradient of Pt NP dilutions, measured using UV-Vis spectroscopy, and (c) average mass of Pt NPs deposited on the electrode samples after DC- and PDC-EPD (N = 7).

S3. Force and energy generated during stability tests

Agarose gel test:

Frictional force during the agarose gel test was calculated using Stoke's law (Equation 1). Here, the sample radius was 38 µm, the viscosity of agarose gel was 5.6 Pa.s[2], and the manually measured push-pull rate was 1.33 mm s^{-1}.

$$F = 6\pi \cdot r \cdot \eta \cdot v \quad (1)$$

Upon calculation, the friction force was equal to **5.35 µN**.

Ultrasonication test:

Energy developed during ultrasonic agitation was calculated using Equation 2.

$$E = P \cdot t \quad (2)$$

Here, the power (P) of the ultrasonicator was 2880 W[3] and time (t) is equal to 300 s. The calculated energy applied to the samples was **864 kJ**.

S4. Mechanical stability of DC-coated Pt-Ir samples

Figure S3(a,b,c) shows exemplary cyclic voltammograms of DC-coated samples, before and after the stability tests: dipping in agarose gel, adhesion test according to ASTM D3359–17, and ultrasonication for 5 min in Milli-Q water. Figure S3(d) shows the average electrochemical surface areas (ECSA) and Figure S3(e) shows the charge storage capacities (CSC) of the samples, before and after stability tests. It can be seen that, since DC-EPD produces more agglomerated deposits[1], upon stability testing all the samples showed a significant reduction in ECSA. However, the least amount of disintegration was seen in agarose gel (20% ECSA decrease). This could be because the clustered deposits are loosely bound to one another and are easily removable by ultrasonic waves. If the adhesive tape would have stuck onto an agglomerated deposit, it also in turn removed a high number of

particles from the surface.

Figure S4(a,b,c) shows exemplary electrochemical impedance (EIS) spectra of DC-coated samples, before and after the stability tests. Figures S4(d) and (e) show the average impedance values at 150 Hz and 1 kHz respectively, before and after the tests. Since the impedance and ECSA are inversely proportional to each other, the EIS analysis conforms with the ECSA analysis, where the ultrasonication shows the highest statistically significant increase (264% at 150 Hz and 98% at 1 kHz) in impedance and the agarose gel test shows the least increase (13% at 150 Hz and 26% at 1 kHz), which is significant at 1 kHz. Figure S5 shows exemplary SEM images of the Pt-Ir surfaces before and after stability tests. It should be noted that the SEM analysis suffers from bad statistics and it is difficult to image the same area before and after the test. Nevertheless, the after-test images still reveal the presence of NPs on them confirming that in no testing method, the NPs are completely removed.

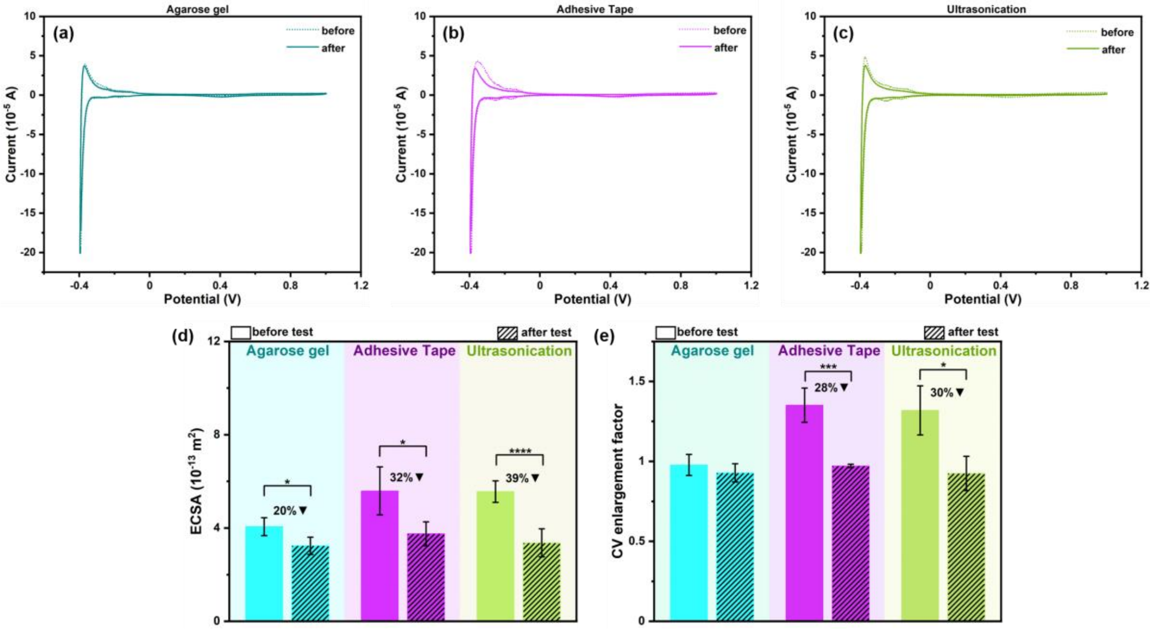

Figure S3: (a,b,c) Exemplary cyclic voltammograms of DC-coated Pt-Ir wires, before and after stability tests. Average (d) ECSA (N = 4, α = 0.05) and (e) CV enlargement factor (CSC, N = 3, α = 0.05) of the DC-coated samples before and after the stability tests.

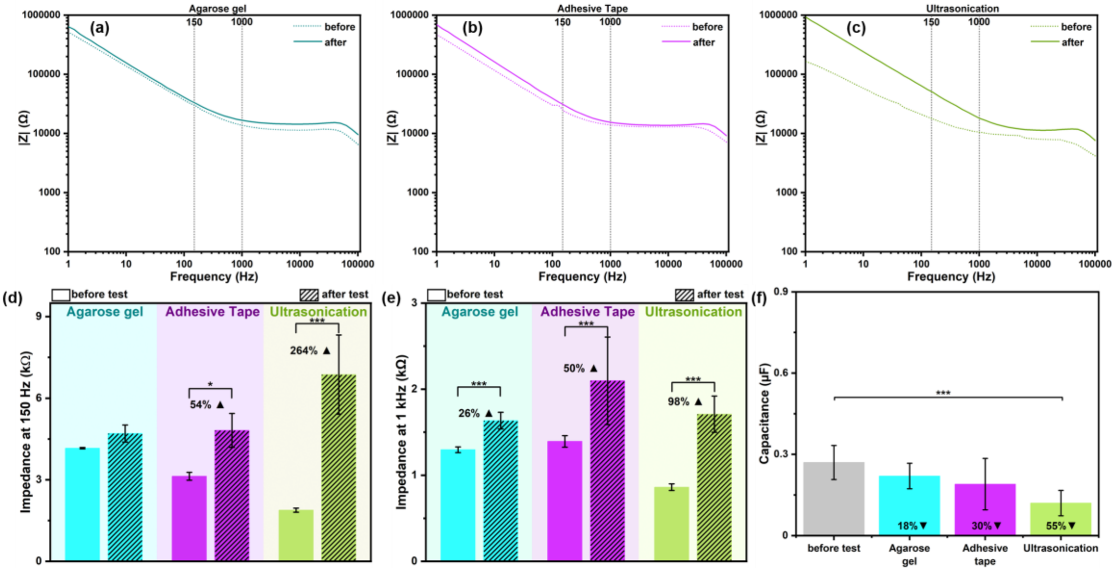

Figure S4: (a,b,c) Exemplary EIS spectra of DC-coated Pt-Ir wires, before and after stability tests. Average impedance values at (d) 150 Hz (N = 4, α = 0.05), (e) 1 kHz (N = 4, α = 0.05). (f) Average capacitance values of the DC-coated samples before and after the stability tests, showing significant capacitance reduction after ultrasonication (N = 3, α = 0.05).

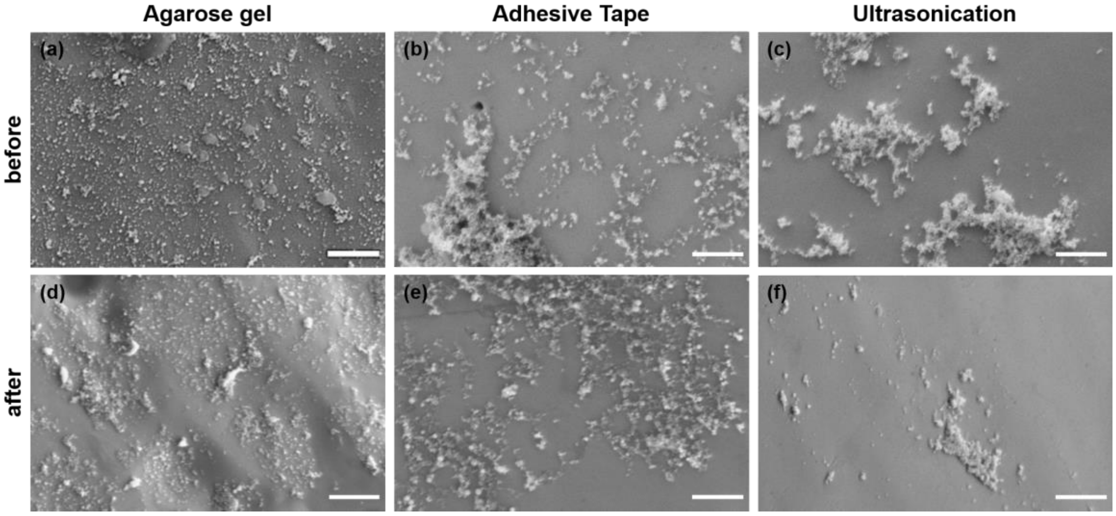

Figure S5: Exemplary SEM images of the DC-coated samples (a,b,c) before and (d,e,f) after the stability tests. Scale bars are 500 nm.

S5. Cyclic voltammograms and EIS spectra of PDC-coated samples

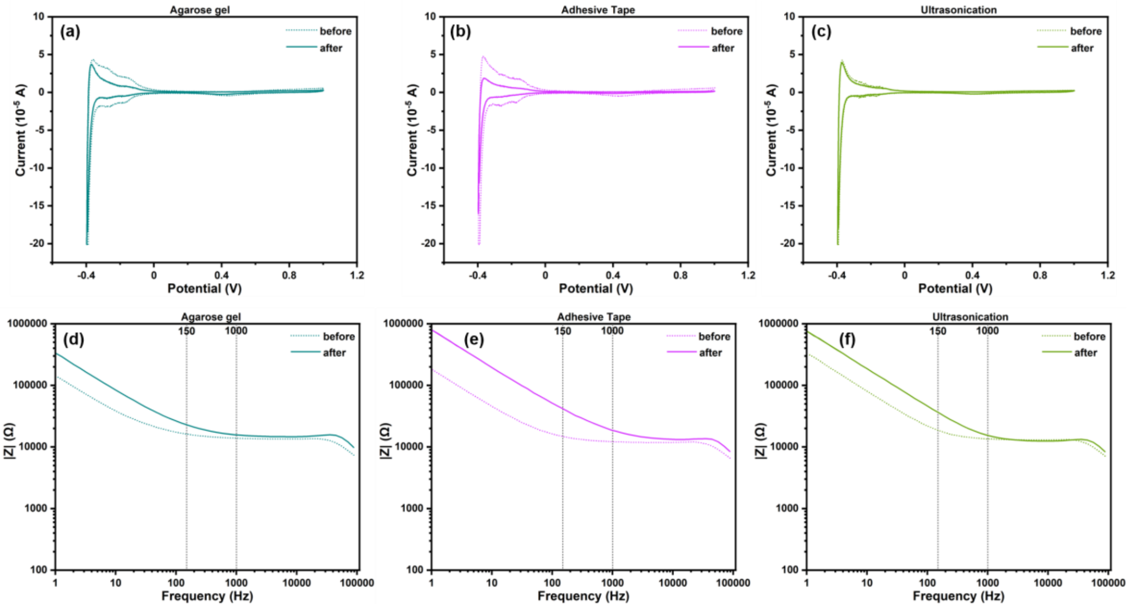

Figure S6: (a,b,c) Exemplary cyclic voltammograms of PDC-coated Pt-Ir wires, before and after stability tests, and (d,e,f) exemplary EIS spectra of PDC-coated Pt-Ir wires, before and after stability tests.

S6. EIS analysis of electrochemical stability tested samples

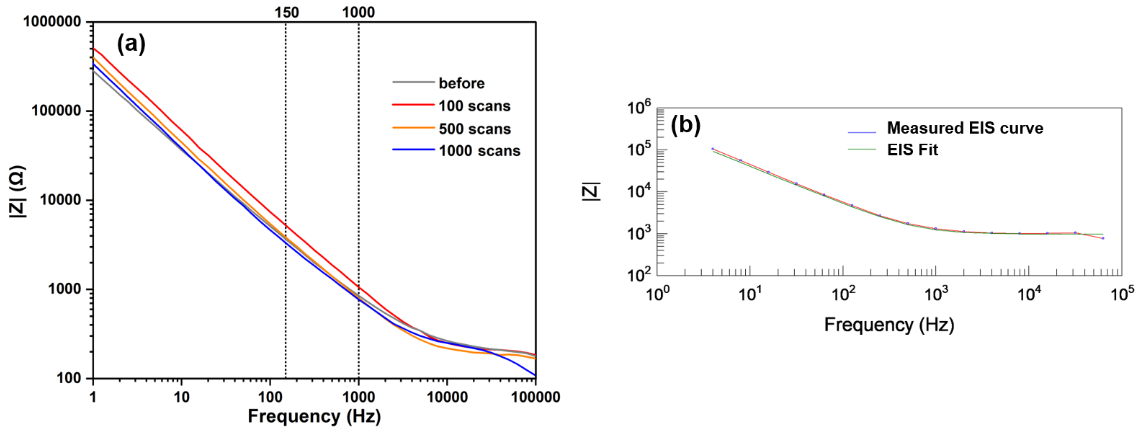

Figure S7: (a) Exemplary EIS spectra of PDC-coated Pt-Ir wires, before and after electrochemical stability tests, and (b) an exemplary EIS fit performed on one of the measured spectra.

S7. LA-ICP-MS controls with rat brain sections

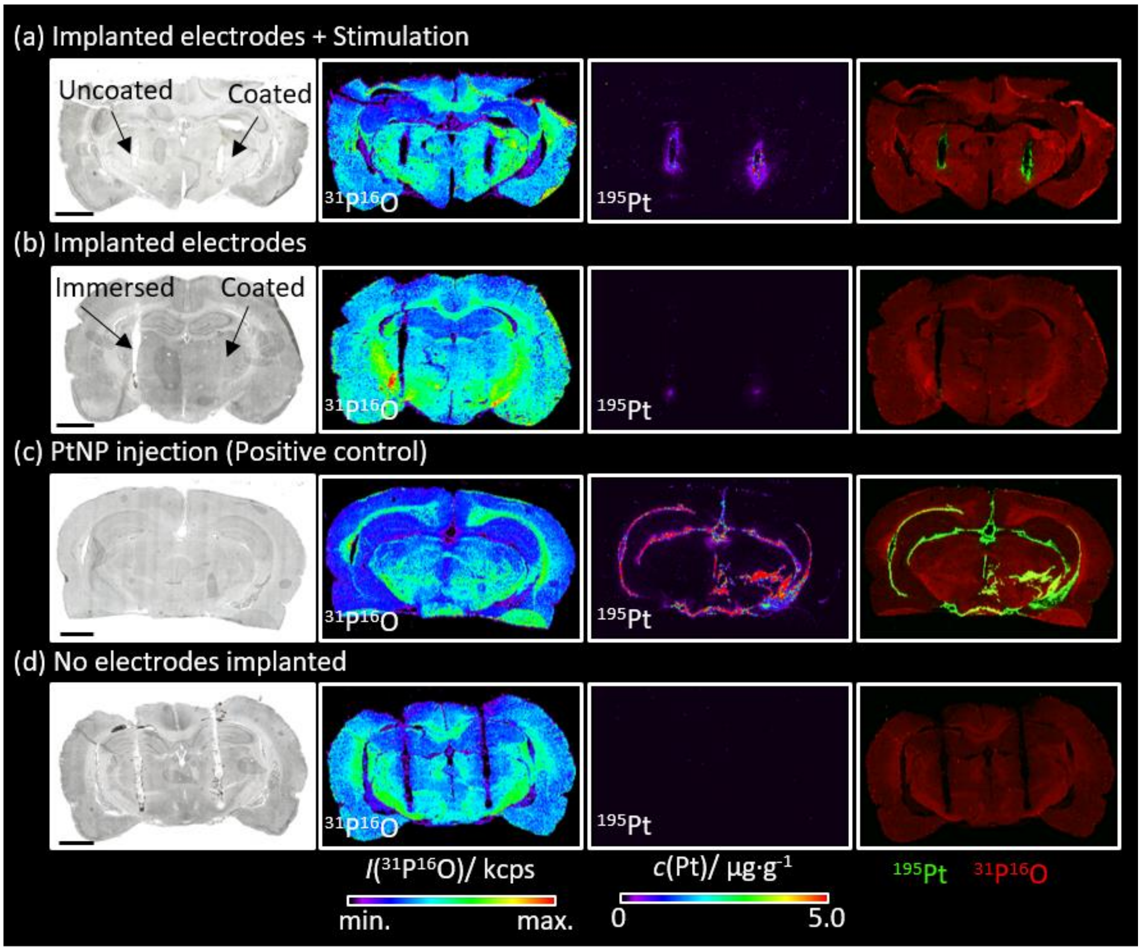

Figure S8: Pt biodistribution in rat brain sections visualized by LA-ICP-MS: (a) Brain section stimulated with uncoated and PDC-coated electrodes. (b) Brain section with implantation of immersed and PDC-coated electrodes removed after one hour without stimulation. (c) Brain section after injection of Pt NPs. (d) Brain section without electrode implantation. Shown are the optical microscopic images, LA-ICP-MS distribution

images of $^{31}P^{16}O$ and ^{195}Pt as well as overlay images of $^{31}P^{16}O$ (red) and ^{195}Pt (green). The scale bar indicates 2 mm.

S8. Bending Assay

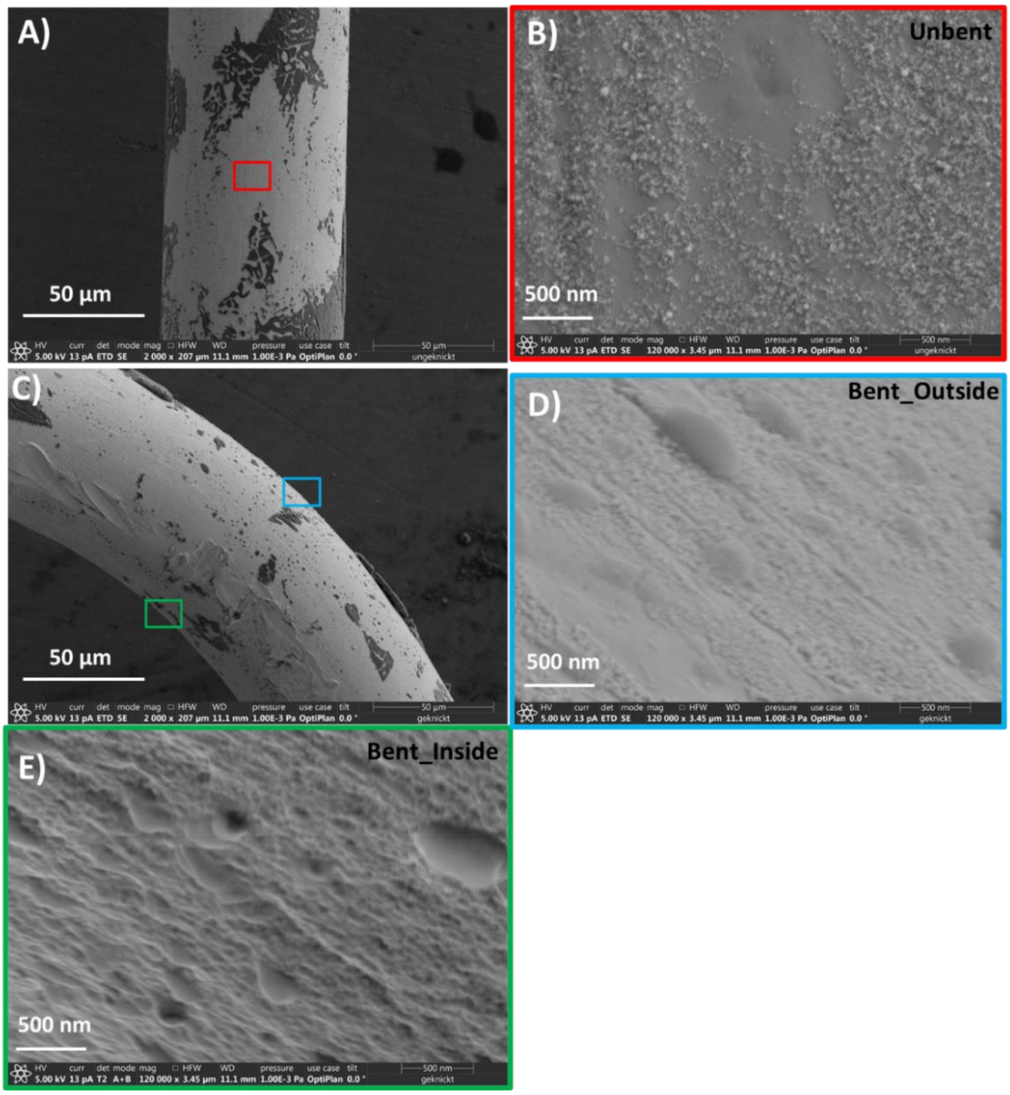

Figure S9: Bending Assay. A) Coated Pt-Ir wire (unbent control) 2,000x magnification. B) Coated Pt-Ir wire (unbent control) 120,000 x magnification. C) Coated Pt-Ir wire bent by ~90° 2,000 x magnification. D) Coated Pt-Ir wire bent by ~90°, Outside, 120,000 x magnification. E) Coated Pt-Ir wire bent by ~90°, Inside, 120,000 x magnification.

References

[1] Ramesh et al. Comparing Direct and Pulsed-Direct Current Electrophoretic Deposition on Neural Electrodes: Deposition Mechanism and Functional Influence. *Langmuir*, 2021, 37(32), 9724-9734.

[2] Fallenstein et al. Dynamic mechanical properties of human brain tissue. *Journal of Biomechanics*, 2.3, 1969, 217-226.

[3] Allpax GmbH & Co. KG, ‘‘https://www.allpax.de/index.php/cat/c22227_Ultraschallreinigungsgeraete-Standard.html’’, 14.09.2021.
